# Recent intake of direct oral anticoagulants and acute ischemic stroke: real world data from a comprehensive stroke center

**DOI:** 10.1186/s42466-025-00438-4

**Published:** 2025-10-29

**Authors:** Doreen Pommeranz, Nicole Lehr, Jordi Kühne Escolà, Bastian Brune, Philipp Dammann, Yan Li, Cornelius Deuschl, Michael Forsting, Clemens Kill, Christoph Kleinschnitz, Martin Köhrmann, Benedikt Frank

**Affiliations:** 1 Department of Neurology and Center for Translational Neuro- and Behavioral Sciences (C-TNBS), University Hospital Essen, Hufelandstr. 55, 45147 Essen, Germany; 2 Department of Trauma, Hand and Reconstructive Surgery, University Hospital Essen, Essen, Germany; 3 Medical Emergency Service of the City of Essen, Essen, Germany; 4 Department of Neurosurgery and Spine Surgery, University Hospital Essen, Essen, Germany; 5 Institute of Diagnostic and Interventional Radiology and Neuroradiology, University Hospital Essen, Essen, Germany; 6 Center of Emergency Medicine, University Hospital Essen, Essen, Germany

**Keywords:** Direct oral anticoagulants, Anticoagulation testing, DOAC plasma levels, Anti-factor Xa activity, Acute ischemic stroke

## Abstract

**Background:**

Deciding on intravenous thrombolysis (IVT) in acute ischemic stroke (AIS) patients with reported recent direct oral anticoagulant (DOAC) intake remains challenging due to concerns about hemorrhagic risk and the absence of randomized controlled trial evidence. This study aimed to provide a comprehensive characterization of all AIS patients with reported recent DOAC intake—regardless of IVT eligibility—treated at a comprehensive stroke center that routinely measures calibrated anti-facor IIa/Xa activity at admission.

**Methods:**

In this retrospective study, clinical and procedural data from AIS patients with recent DOAC intake and calibrated anti-factor IIa/Xa activity measured within three hours of admission were analyzed. Patients were treated at the University Hospital Essen between March 2017 and October 2023.

**Results:**

Among 469 included patients, anti-factor IIa/Xa activity was ≤ 30 ng/ml in 28%, > 30– ≤ 50 ng/ml in 9%, > 50– ≤ 75 ng/ml in 9%, > 75– ≤ 100 ng/ml in 9% and > 100 ng/ml in 45%. Lower DOAC levels correlated with severe stroke symptoms at admission (*ρ* = − 0.263, *p* < 0.001). IVT was administered to 33.5% of patients with DOAC levels ≤ 50 ng/ml, compared to only 4% among those with levels > 50 ng/ml, the majority of whom received prior reversal with idarucizumab. Symptomatic intracranial haemorrhage (sICH) occurred in 4% of IVT-treated and 1% of non-IVT-treated patients, without association to anticoagulation status.

**Conclusions:**

A considerable proportion of AIS patients with recent DOAC intake exhibited minimal or no anticoagulant activity at presentation. Those with the lowest levels also showed highest stroke severity. IVT was safe across all DOAC level groups, with low and comparable sICH rates. These findings support the rationale for a randomized trial evaluating IVT without prior DOAC level testing.

**Supplementary Information:**

The online version contains supplementary material available at 10.1186/s42466-025-00438-4.

## Background

Direct oral anticoagulants (DOACs) have shown non-inferiority or superiority to vitamin K antagonists (VKA) in preventing thromboembolic events, with a more favourable bleeding profile [1–5]. As a result, their use for stroke prevention in patients with non-valvular atrial fibrillation (NVAF) has steadily increased [6]. Fixed dosing regimens, fewer dietary and drug interactions and no requirement of drug monitoring are assumed to enhance therapy adherence. Despite these advantages, 1–2% of patients on DOACs for NVAF experience an acute ischemic stroke (AIS) each year [7, 8]. Among patients eligible for intravenous thrombolysis (IVT), 10–20% are on DOACs [9–11]. Although DOAC-treated patients were excluded from pivotal thrombolysis trials, a growing body of observational data—now exceeding that for other scenarios such as Vitamin K antagonists (VKA) [12, 13] or Dual Antiplatelet Therapy (DAPT) [14]—supports the safety of IVT in carefully selected cases. Nevertheless, according to current European Stroke Organisation (ESO) guidelines, DOAC intake within 48 h prior to stroke onset constitutes a relative contraindication to IVT [15]. Consequently, many patients are still excluded from thrombolysis based solely on reported intake time, despite considerable inter- and intra-individual variability in DOAC pharmacokinetics and pharmacodynamics [16–18]. Calibrated anti-factor IIa/Xa activity assays have emerged as reliable indicators of anticoagulant activity [15, 18]. However, current safety thresholds for IVT eligibility are primarily based on expert opinion and limited pilot data [19]. According to the summaries of product characteristics (SmPC) for Alteplase and Tenecteplase, IVT may be considered if coagulation tests are below the respective upper limit of normal [20]. However, the use of such tests is constrained by feasibility, costs and accessibility, limiting their routine implementation to specific conditions and selected, experienced centers [21, 22].

Given the growing number of AIS patients with recent DOAC intake and the variability in anticoagulation status at presentation, our study aimed to characterize this increasingly relevant patient population in a real-world clinical setting. Specifically, we sought to (1.) report the distribution of DOAC plasma levels at admission in all AIS patients with documented DOAC therapy, (2.) assess associations with stroke severity and outcome and (3.) describe treatment patterns and safety outcomes, particularly in the context of IVT across different anticoagulation strata. Importantly, our cohort was not restricted to patients eligible for reperfusion therapy but reflects the full clinical spectrum of AIS presentations with recent DOAC exposure.

## Methods

### Study design

This retrospective, single-center, observational cohort study was conducted at University Hospital Essen. The study protocol was reviewed and approved by the local ethics committee (18–8408-BO). Due to the retrospective nature of the study, formal patient consent was not required. Nonetheless, all data were handled in accordance with institutional ethical standards, national data protection regulations and the Declaration of Helsinki.

### Patient population and data acquisition

We included data from AIS patients with reported recent intake of apixaban, edoxaban, rivaroxaban or dabigatran who presented to the emergency department between March 2017 and October 2023. Patients were eligible if they met the following criteria:

1. Clinical and/or imaging-based diagnosis of AIS (including transient ischemic attack); computed tomography (CT) was the preferred modality.
2. Documented current DOAC therapy and reported last DOAC intake < 48 h before symptom onset.
3. Measurement of calibrated anti-factor IIa/Xa activity within 3 h of hospital admission.
4. Age ≥ 18 years.

Exclusion criteria were:

1. Time from symptom onset to admission > 72 h
2. Stroke mimic, intracranial hemorrhage (ICH) or any alternative primary diagnosis.

To reflect the acute care setting, we included patients presenting within 72 h after symptom onset. This pragmatic time window was chosen to exclude chronic infarcts or delayed referrals and to focus on patients presenting during the phase in which acute diagnostic and treatment decisions, including those related to anticoagulation, are typically made. Data were sourced from the institutional stroke registry. Laboratory results were exported from the local information system and merged with clinical data. Structured clinical information including the National Institutes of Health Stroke Scale (NIHSS) and the modified Rankin Scale (mRS) was entered by treating neurologists. Follow-up neuroimaging was performed within 24 h after IVT and/or endovascular thrombectomy (ET) or earlier in the event of new or worsening neurological deficits. All imaging was independently reviewed by a neurologist and a neuroradiologist. ICH was classified according to the Heidelberg Bleeding Classification [23]. Symptomatic ICH (sICH) was defined based on ECASS III criteria as a hemorrhage causing neurological deterioration (≥ 4-point increase in NIHSS) or resulting in death [24].

### Laboratory testing

**Fig. 1:**
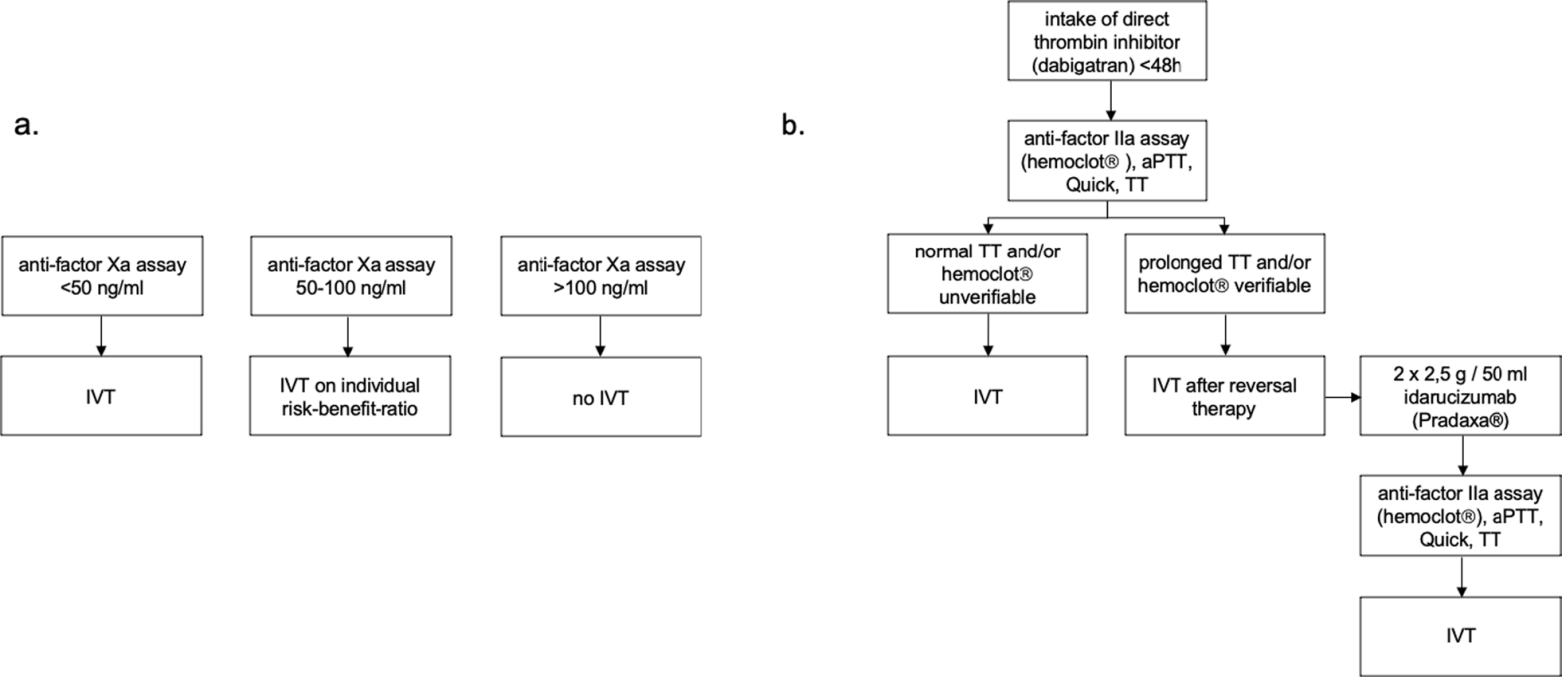
Institutional guidelines for IVT (0.9 mg/kg Alteplase within 4.5 h after symptom onset or perfusion imaging based in the extended time window) under therapy with (**a**) direct factor Xa inhibitors and (**b**) direct thrombin inhibitors. *aPTT* Activated partial thromboplastin time, *IVT* Intravenous thrombolysis, *TT* Thrombin time

At the University Hospital Essen, calibrated anti-factor IIa/Xa activity is routinely measured in all stroke patients reported to be on DOACs. Blood samples for coagulation testing are drawn immediately upon admission, typically in parallel with NIHSS assessment or neuroimaging. To ensure that only initial (‘on-admission’) DOAC plasma levels were analysed, we included measurements performed within the first 3 h after hospital arrival. This threshold was chosen to exclude delayed or follow-up assessments and reflects routine emergency workflows. Regarding our institutional treatment protocol (Fig. 1), IVT with 0.9 mg/kg Alteplase is administered either within 4.5 h after symptom onset or in the extended time window based on CT perfusion imaging, provided that DOAC plasma levels are < 50 ng/ml. For patients on direct factor Xa inhibitors with levels between 50 and 100 ng/ml, IVT is considered based on individualized risk–benefit assessment, with ET preferred as the first-line treatment where appropriate. Patients on the direct thrombin inhibitor dabigatran represent a special subgroup due to the availability of the specific reversal agent idarucizumab. To guide reversal therapy decisions, calibrated anti-factor IIa activity (HEMOCLOT®), activated partial thromboplastin time (aPTT) and thrombin time (TT) are measured. A normal TT—the most sensitive global test—excludes clinically relevant dabigatran activity [25]. In cases of normal TT or undetectable HEMOCLOT®, IVT is administered without reversal. If TT is prolonged or HEMOCLOT® is quantifiable, idarucizumab 2 × 2.5 g is administered, followed by reassessment of HEMOCLOT®, aPTT and TT. IVT is then initiated without additional delay for laboratory confirmation.

### Statistical analysis

All statistical analyses were performed using SPSS Statistics, version 29 (IBM Corp., Armonk, NY, USA). Figures were generated using Microsoft Excel, version 16.89.1 (Microsoft Corp., Redmond, WA, USA). The cohort was stratified into five groups based on commonly referenced DOAC plasma level thresholds. The widely accepted threshold for “safe treatment” with IVT or surgery is < 30 ng/ml [26]. The French Society of Vascular Neurology recommends consideration of IVT at DOAC plasma levels < 50 ng/ml [18], a threshold supported by a German cohort study reporting only one sICH among 261 patients [27]. Prior studies and institutional guidelines often define a DOAC plasma level of 100 ng/ml as the upper threshold for IVT eligibility. Two single-center studies from Basel (Switzerland) and Erlangen (Germany) investigated 18 and 24 AIS patients, respectively, who received IVT despite DOAC plasma levels up to 100 ng/ml. No sICH was observed in the Basel cohort and only one case was reported in the Erlangen cohort [27, 28].

Descriptive statistics were used to summarize demographics, treatment and outcomes. Continuous variables are presented as medians with interquartile ranges (IQR), categorical variables as absolute and relative frequencies. For subgroup analyses, the cohort was dichotomized into patients without anticoagulant activity (anti-factor IIa/Xa activity ≤ 30 ng/ml) and those with anticoagulant activity (anti-factor IIa/Xa activity > 30 ng/ml). Fisher’s exact test and the Mann–Whitney U test were used to compare categorical and continuous variables, respectively. Statistical significance was set at *p* < 0.05 (two-tailed). Additional subgroup analyses compared IVT- and non-IVT-treated patients across DOAC levels. Spearman’s rank correlation was used to assess the association between DOAC level and stroke severity at admission.

## Results

**Fig. 2:**
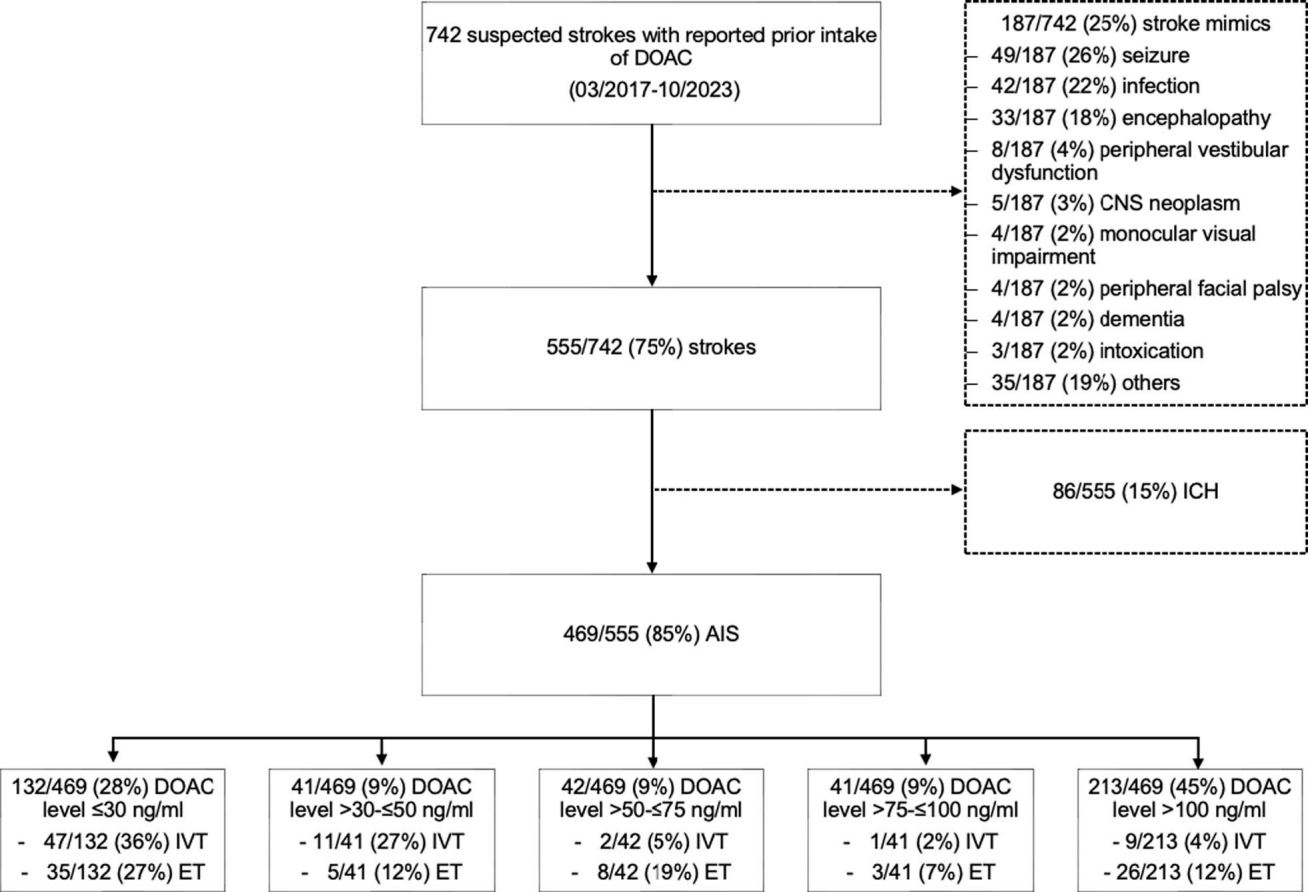
Flowchart of study population inclusion process. *AIS* Acute ischemic stroke, *CNS* Central nervous system, *ET* Endovascular therapy, *ICH* Intracranial hemorrhage, *IVT* Intravenous thrombolysis

During the study period between March 2017 and October 2023, a total of 6108 AIS patients were admitted to the University Hospital Essen. 742 patients with reported recent DOAC intake were admitted due to suspected acute stroke (Fig. 2). Among these, 187 (25%) were diagnosed with stroke mimics, most commonly seizures (26%) and infections (22%). A clinical diagnosis of stroke was confirmed in 555 patients (75%), with neuroimaging identifying ICH in 86 cases (15%), including 22 traumatic and 64 non-traumatic hemorrhages. Ultimately, 469 of 555 patients (85%) were diagnosed with acute ischemia.

Among these 469 AIS patients, DOAC plasma level was ≤ 30 ng/ml in 132 (28%) patients, > 30- ≤ 50 ng/ml in 41 (9%), > 50- ≤ 75 ng/ml in 42 (9%), > 75- ≤ 100 ng/ml in 41 (9%), and > 100 ng/ml in 213 (45%).

### Baseline characteristics

The median age of the cohort was 81 years (IQR 74–86) and 54% were female (Table 1). Comorbidities were common and comparably distributed across DOAC level subgroups. Prior stroke was reported in 50% of patients with DOAC levels > 100 ng/ml (106/213). Renal impairment (serum creatinine ≥ 1.2 mg/dl) was present in 36% of all patients, with no clear association to DOAC level.

**Table 1:** Baseline characteristics, procedural data and outcomes, stratified by DOAC plasma level

	Overall (n = 469)	DOAC level ≤ 30 ng/ml (n = 132, 28%)	DOAC level > 30- ≤ 50 ng/ml (n = 41, 9%)	DOAC level > 50- ≤ 75 ng/ml (n = 42, 9%)	DOAC level > 75- ≤ 100 ng/ml (n = 41, 9%)	DOAC level > 100 ng/ml (n = 213, 45%)
*Demographics and functional status*						
Age (in years)	81 (74–86)	81 (71–86)	80 (70–86)	82 (78–86)	84 (71–88)	81 (75–86)
Females	254 (54%)	77 (58%)	21 (51%)	21 (50%)	21 (51%)	114 (54%)
NIHSS at admission	6 (2–13)	10 (5–18)	6 (1–11)	7 (3–14)	5 (2–12)	4 (2–11)
mRS ≤ 2 prior to admission	276 (59%)	81 (61%)	27 (66%)	26 (62%)	24 (59%)	118 (55%)
*Comorbidities*						
Arterial hypertension	345 (74%)	99 (75%)	30 (73%)	35 (83%)	30 (73%)	151 (71%)
Diabetes	135 (29%)	32 (24%)	10 (24%)	13 (31%)	12 (29%)	68 (32%)
Prior Stroke	206 (44%)	50 (38%)	10 (24%)	22 (52%)	18 (44%)	106 (50%)
Renal insufficiency*	167 (36%)	45 (34%)	11 (27%)	10 (24%)	17 (42%)	84 (39%)
*Indication for DOAC*						
NVAF	423 (90%)	114 (86%)	33 (81%)	41 (98%)	40 (98%)	195 (92%)
Deep vein thrombosis	13 (3%)	4 (3%)	1 (2%)	1 (2%)	1 (2%)	6 (3%)
Pulmonary embolism	15 (3%)	4 (3%)	6 (15%)	-	-	5 (2%)
Peripheral artery disease	4 (0.9%)	3 (2%)	-	-	-	1 (0.5%)
Coagulopathy	12 (3%)	6 (5%)	1 (2%)	-	-	5 (2%)
LAAO/Valvular transplant	2 (0.4%)	1 (0.8%)	-	-	-	1 (0.5%)
*Type of DOAC*						
Apixaban	248 (53%)	58 (44%)	21 (51%)	22 (52%)	22 (54%)	125 (59%)
Dabigatran	39 (8%)	7 (5%)	5 (12%)	5 (12%)	4 (10%)	18 (9%)
Edoxaban	99 (21%)	39 (30%)	8 (20%)	7 (17%)	12 (29%)	33 (16%)
Rivaroxaban	83 (18%)	28 (21%)	7 (17%)	8 (19%)	3 (7%)	37 (17%)
*Blood sample analysis*						
INR	1.09 (1.03–1.19)	1.04 (0.99–1.10)	1.04 (1.00–1.11)	1.09 (1.04–1.17)	1.10 (1.03–1.18)	1.15 (1.06–1.32)
aPTT	26.10 (23.80–29.15)	23.70 (22.20–25.60)	26.00 (23.90–28.00)	25.6 (24.00–28.90)	26.30 (24.10–28.70)	28.00 (25.65–31.65)
*Procedural data*						
DTN (in minutes)**	53 (40–72)	50 (40–73)	54 (47–72)	65 (-)	57 (-)	52 (33–96)
DTGP (in minutes)	67 (46–86)	75 (55–97)	30 (21–43)	78 (40–114)	65 (-)	66 (51–80)
Length of hospital stay (in days)	4 (2–6)	6 (4–10)	4 (3–5)	5 (3–7)	4 (1–5)	4 (3–8)
*Therapy*						
IVT	70 (15%)	47 (36%)	11 (27%)	2 (5%)	1 (2%)	9 (4%)
ET	77 (16%)	35 (27%)	5 (12%)	8 (19%)	3 (7%)	26 (12%)
PPSB	2 (0.4%)	1 (0.8%)	-	1 (2%)	-	-
Idarucizumab	9 (2%)	2 (2%)	2 (5%)	–	–	5 (2%)
*Outcomes*						
NIHSS at discharge	3 (1–9)	5 (2–20)	3 (1–9)	4 (2–13)	3 (1–7)	3 (1–7)
ΔNIHSS***	1 (0–4)	1 (-2–5)	0 (-1–4)	1 (0–4)	1 (0–5)	1 (0–3)
mRS > 2 at discharge	281 (60%)	92 (70%)	21 (51%)	28 (67%)	21 (51%)	120 (56%)
ICH	18 (4%)	5 (4%)	2 (5%)	2 (5%)	2 (5%)	7 (3%)
- Initial imaging	3 (0.6%)	1 (0.8%)	-	–	–	2 (0.9%)
- Follow-up imaging****	15 (3%)	4 (3%)	2 (5%)	2 (5%)	2 (5%)	5 (2%)
sICH	8 (2%)	2 (2%)	2 (5%)	1 (2%)	–	3 (1%)
In-hospital mortality	59 (13%)	27 (20%)	6 (15%)	7 (17%)	2 (5%)	17 (8%)

Data are n (%) or median (IQR)

^*^renal insufficiency = serum creatinine ≥ 1.2 mg/dl

^**^one missing due to externally performed IVT

^***^ΔNIHSS = NIHSS at admission—NIHSS at discharge

^****^first sight of ICH

The predominant indication for DOAC therapy was NVAF in 90% of cases, followed by pulmonary embolism (3%) and deep vein thrombosis (3%). Apixaban was the most frequently used agent (53%), followed by edoxaban (21%), rivaroxaban (18%) and dabigatran (8%).

**Fig. 3:**
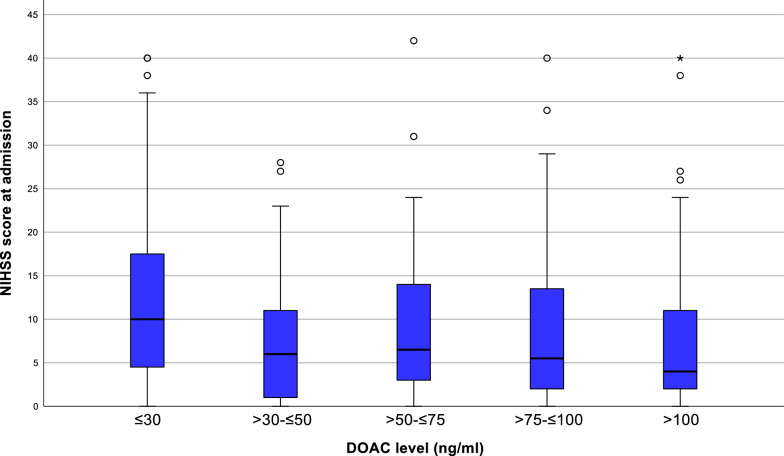
Stroke severity at admission across DOAC plasma level groups. Boxplots displaying NIHSS scores at admission stratified by DOAC plasma level groups (≤ 30 ng/ml, > 30– ≤ 50 ng/ml, > 50– ≤ 75 ng/ml, > 75– ≤ 100 ng/ml, and > 100 ng/ml). Boxes represent interquartile ranges (IQR), horizontal lines indicate medians, whiskers denote 1.5 × IQR and circles represent outliers

Stroke severity at admission, measured by the NIHSS, had a median value of 6. A significant inverse correlation was observed between DOAC plasma levels and NIHSS scores (Spearman’s ρ = − 0.263, *p* < 0.001), indicating that lower anticoagulation levels were associated with more severe strokes (Fig. 3).

### Procedural data

The median time from symptom onset to hospital admission was 2 h and 51 min (IQR 8 h and 5 min). 281 of 469 patients (60%) presented within 4.5 h after symptom onset. Among them, 56 patients (20%) received IVT—a substantially higher than the overall IVT rate (70/469, 15%). The ET rate ranged from 7 to 27% across DOAC level groups, while IVT administration showed a near-linear decline with increasing DOAC levels (Tables 1 and 2).

**Table 2:** IVT-treated patients compared to non-IVT-treated patients, stratified by DOAC plasma level

	DOAC level ≤ 30 ng/ml (n = 132, 28%)		DOAC level > 30- ≤ 50 ng/ml (n = 41, 9%)		DOAC level > 50- ≤ 75 ng/ml (n = 42, 9%)		DOAC level > 75- ≤ 100 ng/ml (n = 41, 9%)		DOAC level > 100 ng/ml (n = 213, 45%)	
	IVT (n = 47, 36%)	No IVT (n = 85, 64%)	IVT (n = 11, 27%)	No IVT (n = 30, 73%)	IVT (n = 2, 5%)	No IVT (n = 40, 95%)	IVT (n = 1, 2%)	No IVT (n = 40, 98%)	IVT (n = 9, 4%)	No IVT (n = 204, 96%)
*Demographics and functional status*										
Age (in years)	81 (74–86)	80 (70–86)	76 (73–85)	82 (70–86)	79 (78–79)	82 (77–86)	93	83 (71–88)	78 (76–86)	81 (75–86)
Females	30 (64%)	47 (55%)	2 (18%)	19 (63%)	1 (50%)	19 (48%)	1 (100%)	20 (50%)	3 (33%)	111 (54%)
NIHSS at admission	11 (7–19)	10 (3–17)	6 (4–11)	5 (1–12)	26 (9–42)	6 (2–11)	19	4 (2–12)	9 (6–11)	4 (2–11)
mRS ≤ 2 prior to admission	33 (70%)	48 (56%)	10 (91%)	30 (100%)	-	24 (60%)	1	23 (58%)	6 (67%)	125 (61%)
*Comorbidities*										
Arterial hypertension	35 (74%)	64 (75%)	10 (91%)	20 (67%)	2 (100%)	32 (80%)	1 (100%)	29 (73%)	6 (67%)	144 (71%)
Diabetes	11 (23%)	21 (25%)	4 (36%)	6 (20%)	1 (50%)	12 (30%)	-	12 (30%)	1 (11%)	66 (32%)
Prior Stroke	23 (49%)	27 (32%)	4 (36%)	6 (20%)	1 (50%)	21 (53%)	-	18 (45%)	6 (67%)	99 (49%)
Renal insufficiency*	19 (40%)	26 (31%)	4 (36%)	7 (23%)	1 (50%)	9 (23%)	1 (100%)	16 (40%)	5 (56%)	78 (38%)
*Indication for DOAC*										
NVAF	39 (83%)	75 (88%)	8 (73%)	25 (83%)	2 (100%)	39 (98%)	1	38 (95%)	8 (89%)	185 (91%)
Deep vein thrombosis	2 (4%)	2 (2%)	-	1 (3%)	-	1 (3%)	-	1 (3%)	-	4 (2%)
Pulmonary embolism	2 (4%)	2 (2%)	2 (18%)	4 (13%)	-	-	-	-	1 (11%)	5 (3%)
Peripheral artery disease	2 (4%)	1 (1%)	-	-	-	-	-	-	-	1 (1%)
Coagulopathy	1 (2%)	5 (6%)	1 (9%)	-	-	-	-	-	-	1 (1%)
LAAO/Valvular transplant	1 (2%)	-	-	-	-	-	-	-	-	-
*Type of DOAC*										
Apixaban	22 (47%)	36 (42%)	7 (64%)	14 (47%)	-	22 (55%)	1	21 (53%)	3 (33%)	122 (60%)
Dabigatran	3 (6%)	4 (5%)	2 (18%)	3 (10%)	1 (50%)	4 (10%)	0	4 (10%)	4 (44%)	14 (7%)
Edoxaban	14 (30%)	25 (30%)	1 (9%)	7 (23%)	1 (50%)	6 (15%)	0	12 (30%)	-	33 (16%)
Rivaroxaban	8 (17%)	20 (24%)	1 (9%)	6 (20%)	-	8 (20%)	0	3 (8%)	2 (22%)	35 (17%)
*Blood sample analysis*										
INR	1.04 (0.98–1.09)	1.05 (1.00–1.13)	1.01 (0.98–1.05)	1.05 (1.03–1.14)	1.10 (1.09–1.11)	1.09 (1.04–1.18)	1.10	1.10 (1.03–1.19)	1.21 (1.25–1-32)	1.14 (1.06–1.32)
aPTT	23.60 (22.15–24.90)	23.75 (22.40–25.80)	26.80 (25.10–28.90)	25.75 (22.20–28.00)	29.00 (26.80–31.20)	25.50 (24.00–28.55)	24.10	26.40 (24.30–29.05)	33.00 (25.25–47.20)	27.90 (25.55–31.45)
*Procedural data*										
DTN (in minutes)	51 (40–73)	-	54 (47–72)	-	45 (27–62)	-	57	-	48 (33–72)	-
DTGP (in minutes)	90 (79–105)	66 (26–80)	21 (-)	22 (14–49)	50 (-)	63 (24–66)	-	63 (48–64)	85 (76–143)	63 (28–71)
Length of hospital stay (in days)	7 (5–10)	6 (4–10)	5 (4–7)	3 (2–5)	20 (10–29)	4 (3–7)	4	4 (1–6)	4 (4–10)	4 (3–8)
*Therapies*										
ET	19 (40%)	16 (19%)	1 (9%)	4 (13%)	1 (50%)	7 (18%)	-	3 (8%)	3 (33%)	23 (11%)
PPSB	1 (2%)	-	-	-	-	1 (3%)	-	-	-	-
Idarucizumab	1 (2%)	1 (1%)	2 (18%)	-	-	-	-	-	4 (44%)	1 (1%)
*Outcomes*										
NIHSS at discharge	7 (4–20)	4 (1–19)	2 (2–12)	3 (0–9)	25 (7–42)	3 (2–12)	4	3 (1–7)	3 (2–7)	3 (1–7)
ΔNIHSS	2 (-4–6)	1 (0–4)	1 (-1–5)	0 (-1–3)	1 (0–2)	0 (-0.5–2)	15	1 (0–5)	5 (3–7)	1 (0–3)
				1						
mRS > 2 at discharge	35 (74%)	57 (43%)	5 (45%)	6 (53%)	2 (100%)	26 (65%)	-	21 (53%)	5 (55%)	115 (56%)
ICH	4 (9%)	1 (1%)	1 (9%)	1 (3%)	-	2 (5%)	-	2 (5%)	1 (11%)	6 (3%)
- Initial imaging	1	-	-	-	-	-	-	-	-	2
- Follow-up imaging	3	1	1	1	-	2	-	2	1	4
sICH	2 (4%)	-	1 (9%)	1 (3%)	-	1 (3%)	-	-	-	3 (1%)
In-hospital mortality	11 (23%)	16 (19%)	1 (9%)	5 (17%)	1 (50%)	6 (15%)	-	2 (5%)	-	17 (8%)

According to institutional protocols consistent with European guidelines, 173 patients (37%) had DOAC levels < 50 ng/ml, considered safe for IVT. Additionally, 83 patients (18%) fell within the "gray zone" (50–100 ng/ml), where IVT is considered based on individualized risk assessment. Despite these findings, only 70 patients (15%) received IVT, predominantly those with DOAC levels ≤ 30 ng/ml (n = 47). IVT was rarely used in patients with levels > 30 ng/ml (n = 23) and often preceded by reversal therapy. The median door-to-needle time (DTN) in the cohort was 53 min (IQR 40–72).

### Safety outcomes

ICH was observed in 18 of 469 patients (4%). The most frequent subtype was subarachnoid hemorrhage (SAH, n = 7), followed by hemorrhagic infarction type 1 (HI1, n = 4), hemorrhagic infarction type 2 (HI2, n = 2), parenchymal hematoma type 2 (PH2, n = 2), and intraventricular hemorrhage (IVH, n = 1). Three cases were detected on initial imaging, while the remaining 15 were identified during follow-up.

Symptomatic ICH (sICH) occurred in 8 patients (2%), with no evident association to DOAC level or IVT administration (sICH rate: 3/70 in the IVT group, 5/399 in the non-IVT group). Among 17 patients with DOAC levels > 30 ng/ml who received IVT without reversal, no cases of sICH occurred in those with levels > 50 ng/ml (Table 2). The most frequent type of sICH was SAH (n = 3), classified according to the Heidelberg Bleeding Classification.

### Anticoagulation status

To investigate differences between patients without anticoagulant activity and those with anticoagulant activity (defined as a calibrated anti-factor IIa/Xa activity ≤ 30 ng/ml respectively > 30 ng/ml), a dichotomization of the cohort was performed (Table 3). Demographics, comorbidities, DOAC indication and agent and procedural parameters were similar between groups (all p ≥ 0.05).

**Table 3:** Patients without anticoagulant activity (Calibrated anti-factor IIa/Xa activity ≤ 30 ng/ml) compared to patients with anticoagulant activity (Calibrated anti-factor IIa/Xa activity > 30 ng/ml)

	Overall (n = 469)	DOAC level ≤ 30 ng/ml (n = 132, 28%)	DOAC level > 30 ng/ml (n = 337, 72%)	*p*-value
*Demographics and functional status*				
Age (in years)	81 (74–86)	81 (70–86)	81 (74–87)	0.404
Females	254 (54%)	77 (58%)	177 (53%)	0.260
NIHSS at admission	6 (2–13)	10 (5–18)	5 (2–11)	< 0.001
mRS ≤ 2 prior to admission	276 (59%)	81 (61%)	195 (58%)	0.664
*Comorbidities*				
Arterial hypertension	345 (74%)	99 (75%)	246 (73%)	0.727
Diabetes	135 (29%)	32 (24%)	103 (31%)	0.212
Prior Stroke	206 (44%)	50 (38%)	156 (46%)	0.121
Renal insufficiency*	167 (36%)	45 (34%)	122 (36%)	0.748
*Indication for DOAC*				
NVAF	423 (90%)	114 (86%)	309 (92%)	0.070
Deep vein thrombosis	13 (3%)	4 (3%)	9 (3%)	1.000
Pulmonary embolism	15 (3%)	4 (3%)	11 (3%)	1.000
Peripheral artery disease	4 (0.9%)	3 (2%)	1 (0.3%)	0.192
Coagulopathy	12 (3%)	6 (5%)	6 (2%)	0.138
LAAO/Valvular transplant	2 (0.4%)	1 (0.8%)	1 (0.3%)	0.281
*Type of DOAC*				
Apixaban	248 (53%)	58 (44%)	190 (56%)	0.006
Dabigatran	39 (8%)	7 (5%)	32 (9%)	0.271
Edoxaban	99 (21%)	39 (30%)	60 (18%)	0.005
Rivaroxaban	83 (18%)	28 (21%)	55 (16%)	0.230
*Blood sample analysis*				
INR	1.09 (1.03–1.19)	1.04 (0.99–1.10)	1.11 (1.04–1.22)	< 0.001
aPTT	26.10 (23.80–29.15)	23.70 (22.20–25.60)	27.20 (24.90–30.80)	< 0.001
*Procedural data*				
DTN (in minutes)	53 (40–72)	50 (40–73)	55 (43–72)	0.660
DTGP (in minutes)	67 (46–86)	75 (55–97)	65 (41–76)	0.073
Length of hospital stay (in days)	4 (2–6)	6 (4–10)	4 (3–7)	< 0.001
*Therapy*				
IVT	70 (15%)	47 (36%)	23 (7%)	< 0.001
ET	77 (16%)	35 (27%)	42 (12%)	< 0.001
PPSB	2 (0.4%)	1 (0.8%)	1 (0.3%)	0.484
Idarucizumab	9 (2%)	2 (2%)	7 (2%)	1.000
*Outcomes*				
NIHSS at discharge	3 (1–9)	5 (2–20)	3 (1–7)	< 0.001
ΔNIHSS	1 (0–4)	1 (-2–5)	1 (0–3)	0.522
mRS > 2 at discharge	282 (60%)	92 (70%)	190 (56%)	0.009
ICH	18 (4%)	5 (4%)	13 (4%)	1.000
sICH	8 (2%)	2 (2%)	6 (2%)	1.000
In-hospital mortality	59 (13%)	27 (20%)	32 (9%)	0.002

Patients without anticoagulant activity presented with significantly more severe stroke symptoms at admission (*p* < 0.001) and were more likely to experience longer hospital stays (*p* < 0.001), higher in-hospital mortality (*p* < 0.001) and worse functional outcome (*p* = 0.009). In contrast, no significant difference was observed in the prevalence of sICH (*p* = 1.000). Although global coagulation parameters are not specific for DOAC activity, both INR and aPTT were significantly higher in patients with higher DOAC levels (*p* < 0.001).

## Discussion

The key findings of this study are: (1.) a substantial proportion of AIS patients with reported recent DOAC intake indicated no anticoagulant activity at the time of admission; (2.) IVT was rarely performed despite laboratory eligibility; and (3.) low DOAC plasma levels were associated with more severe strokes, yet sICH rates remained low across all subgroups.

Approximately one-third of patients with reported recent DOAC intake showed no anticoagulant activity at admission. Despite the routine availability of quantitative DOAC testing at our center, the proportion of patients receiving IVT was comparatively low. This finding may in part be explained by the inclusion of non-eligible patients, such as those presenting with established infarct demarcation, since patients were enrolled up to 72 h after symptom onset. Nevertheless, 60% were admitted within 4.5 h after symptom onset, so treatment decisions frequently reflected a conservative approach. This may be rooted in the historical exclusion of DOAC-treated patients from pivotal IVT trials. Recent observational studies involving several thousand DOAC-treated cases increasingly suggest that IVT can be performed safely in carefully selected patients [29–32], particularly when decisions are informed by calibrated anti-factor IIa/Xa assays [10, 33]. Notably, our results align with these data in showing no increased risk of sICH in patients with DOAC levels > 30 ng/ml. Rates of sICH were comparable between patients without (2%) and patients with anticoagulant activity (2%) and between IVT-treated (4%) and non-IVT-treated (1%) groups. Among 17 patients indicating DOAC levels > 30 ng/ml who underwent IVT without reversal, only one developed sICH. Furthermore, patients with low DOAC levels had significantly higher stroke severity, increased in-hospital mortality and poorer functional outcomes, aligning with previous reports that adequate DOAC therapy mitigates stroke severity without significantly elevating bleeding risk [9, 29, 34].

The median DTN of 53 min in our cohort represents a substantial delay compared to our institutional benchmark of 30 min [35]. This prolongation was primarily attributable to the turnaround time of ~ 30 min for DOAC plasma level testing. The longest DTN were observed in patients with DOAC levels 50–75 ng/ml—a range associated with therapeutic ambiguity and heightened medicolegal caution. Interestingly, a delay in IVT administration was observed in dabigatran-treated patients, despite clear recommendations in our institutional protocol to proceed with immediate IVT following idarucizumab administration without waiting for baseline plasma levels or its reduction. This discrepancy likely reflects a limited familiarity with idarucizumab use in the hyperacute stroke setting during the earlier part of the study period, as well as medicolegal caution among treating physicians. While quantitative testing improves safety, it may delay urgent interventions. Standardized workflows and increased familiarity with reversal protocols could mitigate these delays. Additionally, alternative laboratory methods, such as urine dipstick tests, modified thromboelastography with anti-Xa assays and ecarin clotting time-based cartridges, offer the potential for a more rapid and widely accessible assessment of the anticoagulation status [19, 36].

### Limitations and future directions

This study has limitations inherent to retrospective analyses. As a single-center observational study, causal inferences cannot be drawn. Furthermore, all IVT treatments in our cohort were performed exclusively with Alteplase. Since the completion of data collection, our center—like many others—has initiated a transition toward Tenecteplase [37]. As a result, the generalizability of our findings to current practice, in which Tenecteplase is increasingly used as the standard thrombolytic agent, may be limited. The use of a specific reversal agent was limited to idarucizumab in patients with prior dabigatran exposure. During the study period, andexanet alfa was not used due to its conditional approval status, limited availability and unresolved safety concerns, particularly in the context of IVT [38, 39]. Therefore, management of patients on factor Xa inhibitors was guided exclusively by quantitative plasma level assessment.

Our findings raise important questions regarding DOAC pharmacodynamics and treatment response. In a notable proportion of patients, plasma levels were below the anticipated therapeutic threshold, despite ongoing prescription. Due to the study design, we were unable to reliably determine whether these low or undetectable levels were the result of poor adherence or represented a pharmacokinetic or pharmacodynamic failure (i.e. true non-responders). Structured assessment of medication adherence was not performed, as patient self-reporting in the acute setting is often unreliable. Although formal DOAC challenge testing, i.e. measurement of trough and peak plasma levels after standardized intake, is available at our institution, it is reserved for specific clinical scenarios. In clinical practice, we more commonly switch to an alternative anticoagulant rather than re-expose patients to the same DOAC under controlled conditions. These uncertainties highlight the potential value of routine DOAC level testing after treatment initiation or re-initiation, particularly in high-risk individuals, to identify non-responders and guide personalized anticoagulation strategies.

## Conclusions

A substantial proportion of AIS patients with reported recent DOAC use showed no anticoagulant activity at hospital admission. Nevertheless, IVT was underutilized reflecting persistent uncertainty in clinical practice. Importantly, use of IVT was not associated with increased sICH risk in patients with DOAC levels > 30 ng/ml. Our findings support the rationale for a randomized controlled trial evaluating IVT in AIS patients with reported DOAC use and without prior DOAC level testing.

## Supplementary Information

Supplementary Material 1.

## Data Availability

The datasets used and analyzed for the current study are available from the corresponding author on reasonable request.

## Abbreviations

AIS: Acute ischemic stroke
aPTT: Activated partial thromboplastin time
CT: Computed tomography
DAPT: Dual antiplatelet therapy
DOAC: Direct oral anticoagulant
DTN: Door-to-needle time
ESO: European stroke organisation
ET: Endovascular thrombectomy
HI1: Hemorrhagic infarction type 1
HI2: Hemorrhagic infarction type 2
ICH: Intracranial hemorrhage
IQR: Interquartile ranges
IVH: Intraventricular hemorrhage
IVT: Intravenous thrombolysis
mRS: Modified rankin scale
NIHSS: National institutes of health stroke scale
NVAF: Non-valvular atrial fibrillation
PH2: Parenchymal hematoma type 2
SAH: Subarachnoid hemorrhage
sICH: Symptomatic intracranial hemorrhage
SmPC: Summaries of product characteristics
TT: Thrombin time
VKA: Vitamin K antagonists
