# Supplementary material for: Recent intake of direct oral anticoagulants and acute ischemic stroke: real world data from a comprehensive stroke center
Source: Neurol Res Pract. 2025 Oct 29;7(1):82. doi: 10.1186/s42466-025-00438-4 (PMC12574025; doi:10.1186/s42466-025-00438-4)
Supplement: Supplementary file 1 — Supplementary Material 1. [file 42466_2025_438_MOESM1_ESM.pptx]

## Slide 1
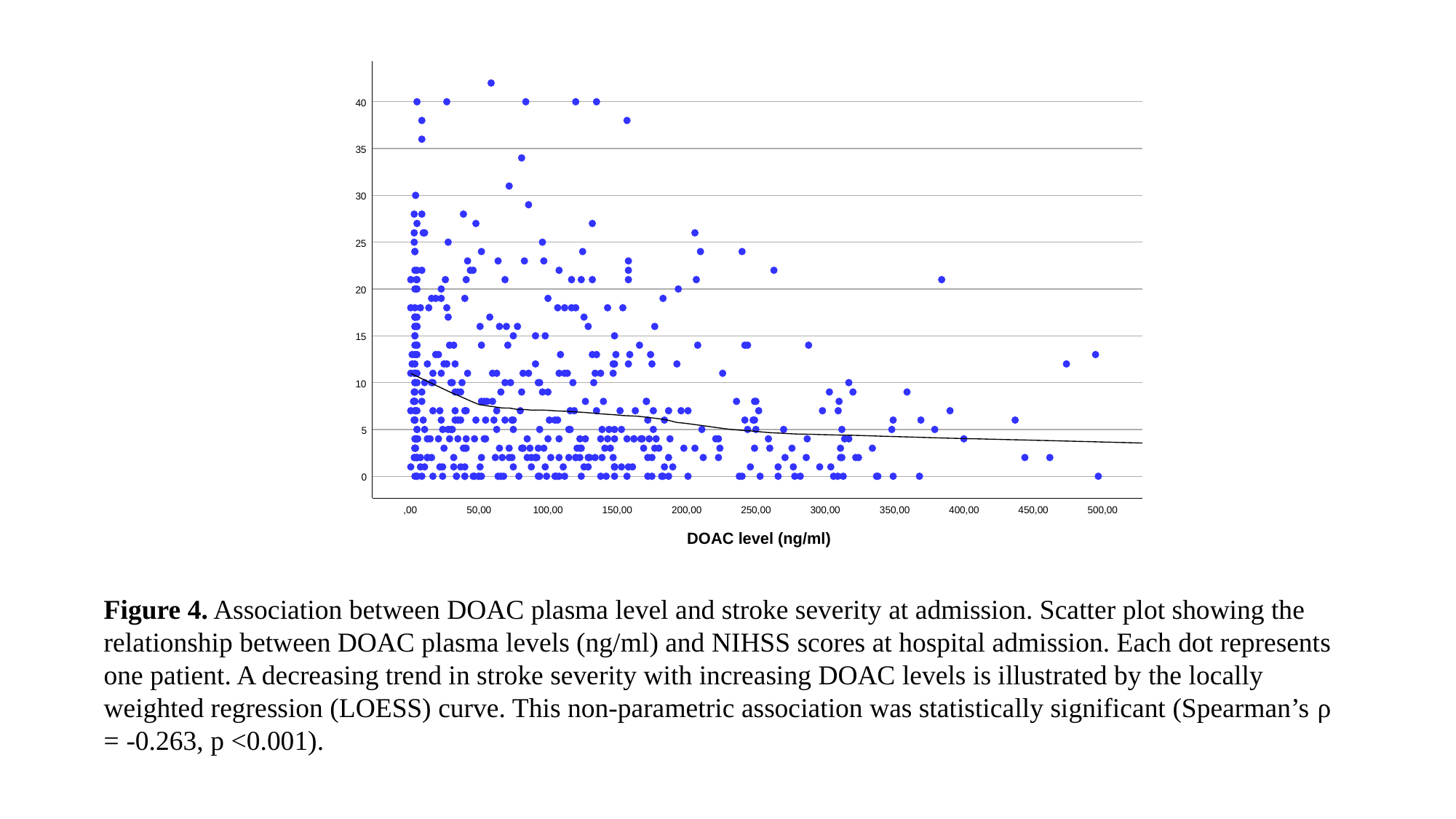

Figure 4. Association between DOAC plasma level and stroke severity at admission. Scatter plot showing the relationship between DOAC plasma levels (ng/ml) and NIHSS scores at hospital admission. Each dot represents one patient. A decreasing trend in stroke severity with increasing DOAC levels is illustrated by the locally weighted regression (LOESS) curve. This non-parametric association was statistically significant (Spearman’s ρ = -0.263, p <0.001).

## Slide 2
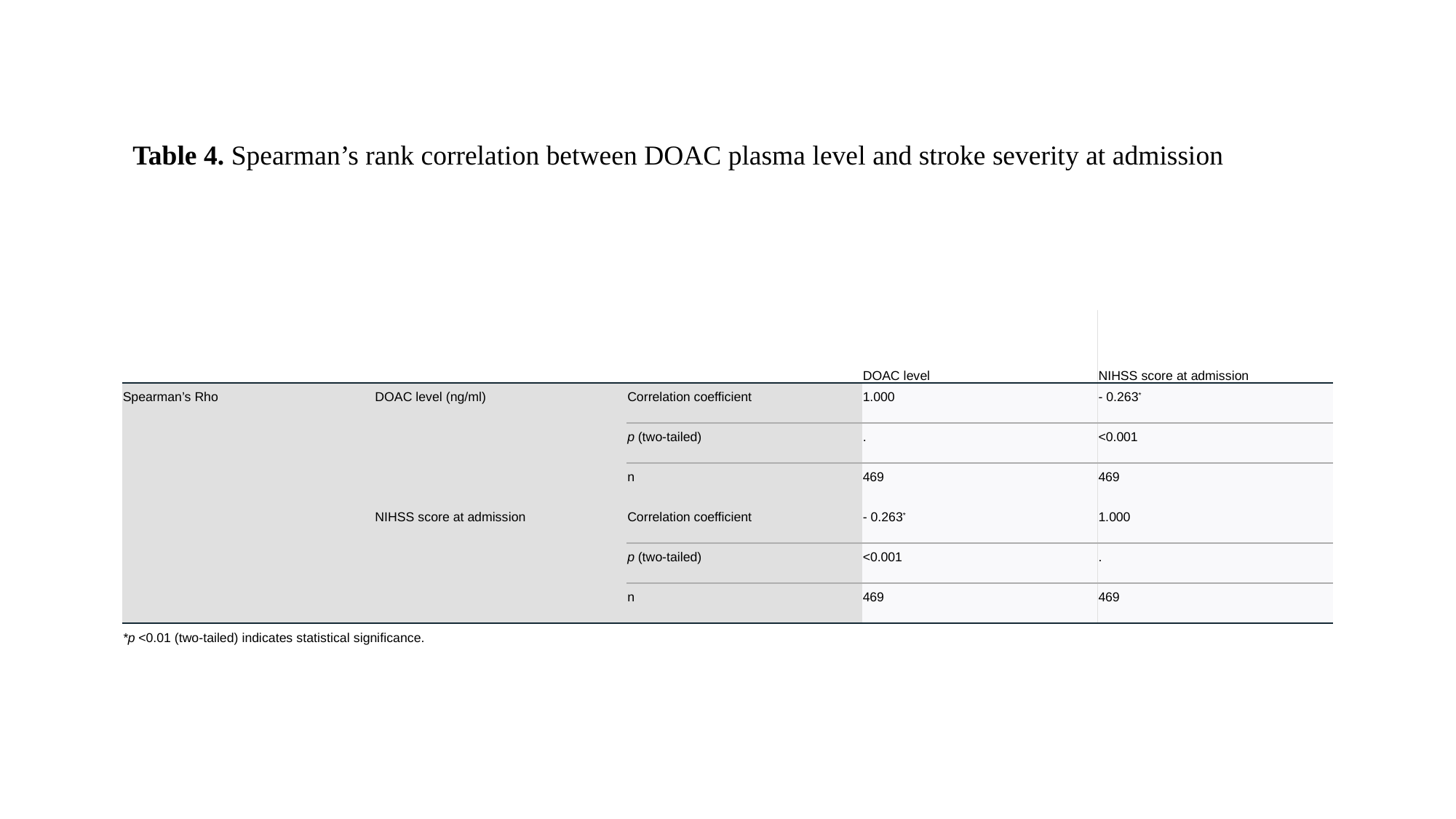

Table 4. Spearman’s rank correlation between DOAC plasma level and stroke severity at admission
| | | | | |
| --- | --- | --- | --- | --- |
| | | | DOAC level | NIHSS score at admission |
| Spearman’s Rho | DOAC level (ng/ml) | Correlation coefficient | 1.000 | - 0.263\* |
| | | p (two-tailed) | . | <0.001 |
| | | n | 469 | 469 |
| | NIHSS score at admission | Correlation coefficient | - 0.263\* | 1.000 |
| | | p (two-tailed) | <0.001 | . |
| | | n | 469 | 469 |
| \*p <0.01 (two-tailed) indicates statistical significance. | | | | |
